# Platelet-rich plasma-enhanced “plum blossom” bone grafting for refractory femoral nonunion: a single-center study of 33 cases

**DOI:** 10.3389/fbioe.2025.1680193

**Published:** 2026-01-29

**Authors:** Jianrong Chen, Shiheng Wang, Huolong Zhou, Zhoulian Feng, Zhiliang Ma, Kunyu Wang, Yuwen Kang

**Affiliations:** 1 The Eighth Clinical Medical College of Guangzhou University of Chinese Medicine, Foshan, Guangdong, China; 2 Foshan Hospital of Traditional Chinese Medicine, Foshan, Guangdong, China; 3 Guangzhou University of Chinese Medicine, Guangzhou, Guangdong, China; 4 Henan University of Chinese Medicine, Zhengzhou, Henan, China

**Keywords:** platelet-rich plasma, nonunion, femoral nonunion, refractory nonunion, recalcitrant nonunion, diamond concept

## Abstract

**Background:**

Revision surgery for femoral nonunion is technically challenging, and there is limited evidence supporting effective treatments for cases with multiple failed revisions. This study aims to evaluate the efficacy of platelet-rich plasma-enhanced “plum blossom” bone grafting combined with a bioactive chamber in treating refractory femoral nonunion after multiple failed revisions.

**Methods:**

A retrospective analysis was conducted on patients with refractory femoral nonunion treated at a high-level trauma center between January 2021 and July 2024. These patients underwent mechanical optimization, platelet-rich plasma -enhanced “plum blossom” autologous iliac bone grafting, and bioactive chamber therapy. Radiographic outcomes included union rate and limb shortening, while clinical outcomes encompassed healing time, visual analog scale pain score, SF-36 quality of life score, Harris hip score, and complications.

**Results:**

Thirty-three patients were included (24 males, 9 females), with a mean age of 42.64 ± 13.03 years. The average number of previous surgeries was 2.64 ± 1.17. The nonunion types were hypertrophic (4 cases, 12.10%), atrophic (24 cases, 72.70%), and oligotrophic (5 cases, 15.20%). The mean bone defect was 3.87 ± 1.05 cm, and the mean follow-up duration was 16.06 ± 3.37 months. The union rate was 96.97% (32/33), with a mean healing time of 9.78 ± 1.75 months. Post-treatment, significant improvements were observed in VAS score (4.27 ± 1.18 vs. 1.21 ± 1.05, p < 0.001), Harris hip score (50.91 ± 8.47 vs. 86.39 ± 7.75, p < 0.001), SF-36 score (59.21 ± 5.63 vs. 84.48 ± 5.32, p < 0.001), and limb shortening (2.20 ± 0.64 vs. 0.32 ± 0.57, p < 0.001). Two patients (6.06%) experienced severe complications (1 case of persistent nonunion, 1 case of deep vein thrombosis).

**Conclusion:**

The synergistic effect of biomechanical stability and biological stimulation is a critical pathway to overcoming traditional treatment limitations. This study provides a reference method for managing refractory and recalcitrant femoral nonunion.

## Introduction

There is currently no universally accepted clinical definition of nonunion (Siverino et al., 2024; Wildemann et al., 2021). Most studies adopt the 1986 FDA definition, which states that nonunion occurs when a fracture fails to heal within 9 months or shows no progress for 3 consecutive months (Wildemann et al., 2021). However, this definition may not apply to all fractures, as healing times vary significantly based on fracture type, injury mechanism, and patient health (Siverino et al., 2024; Wildemann et al., 2021). Currently, diagnosis of nonunion relies on symptoms, physical examination, and imaging (Wildemann et al., 2021).

Treatment strategies for nonunion focus on biomechanical optimization and biological stimulation (Wildemann et al., 2021). Autologous iliac bone grafting is considered the “gold standard,” yet it does not always yield ideal outcomes (Wagner et al., 2024), with success rates ranging from 80% to 93% (Schmidt, 2021). Patients with failed autografts often develop refractory or recalcitrant nonunion, requiring additional interventions (Gagnon et al., 2024; Siverino et al., 2024; Wagner et al., 2024). It has been reported that the incidence rate of refractory femoral fracture nonunion is as high as 16% (Wellings and Blair, 2025). From the initial fracture to the diagnosis of nonunion and subsequent failed revisions, patients often wait at least 1 year, complicating treatment and leading to muscle atrophy, deformity, pain, infection, or dysfunction, severely impacting their physical and psychological wellbeing, particularly in cases of femoral and tibial nonunion (Nicholson et al., 2021; Wildemann et al., 2021). Moreover, due to previous iliac bone grafts, donor sites are limited, leaving clinicians with few effective strategies for such cases. Although there are currently reports on management strategies for failed initial treatment of femoral fractures, few studies have focused on refractory cases that remain ununited after autologous bone grafting. Based on this, the present study conducted a retrospective analysis using the strategy of PRP-enhanced “Plum Blossom” autologous bone grafting combined with internal fixation, aiming to explore its clinical efficacy in patients with multiple revision failures and provide a reference for the treatment of refractory nonunion.

## Study design

### Patients and methods

This single-center retrospective study was approved by the hospital ethics committee (Approval No.: KY[2024]329) and conducted in accordance with the Helsinki Declaration under ethical committee supervision. Refractory nonunion was defined as meeting the basic diagnostic criteria for nonunion (no healing ≥9 months post-surgery and no radiographic progress for 3 consecutive months) despite at least one standardized surgical intervention (including revision with bone grafting and fixation). Patients treated at a high-level trauma center between January 2021 and July 2024 were included.

### Inclusion criteria and exclusion criteria

Inclusion criteria were: 1. meeting the refractory nonunion definition, 2. age ≥18 years, 3. femoral nonunion, 4. previous autograft treatment, 5. PRP-enhanced therapy, and 6. controlled infection (confirmed by CRP, ESR, PCT, and symptoms). Exclusion criteria included: 1. active infection, 2. abnormal platelet count, 3. antiplatelet medication use, 4. follow-up <12 months, 5. loss to follow-up and incomplete medical records, and 6. segmental bone defect (bone defect > 6 cm).

Union criteria were: fracture line blurring on DR imaging, formation of at least three continuous femoral cortical layers, and ability to walk ≥100 m for ≥3 min.

### Outcome measures

Radiographic outcomes included union rate and limb shortening, while clinical outcomes encompassed healing time, VAS score (Åström et al., 2023), SF-36 score (Grönkvist et al., 2024), Harris hip score (Cho et al., 2025), and complications. All patients were diagnosed by a senior trauma orthopedic specialist with over 20 years of experience.

### Technical roadmap


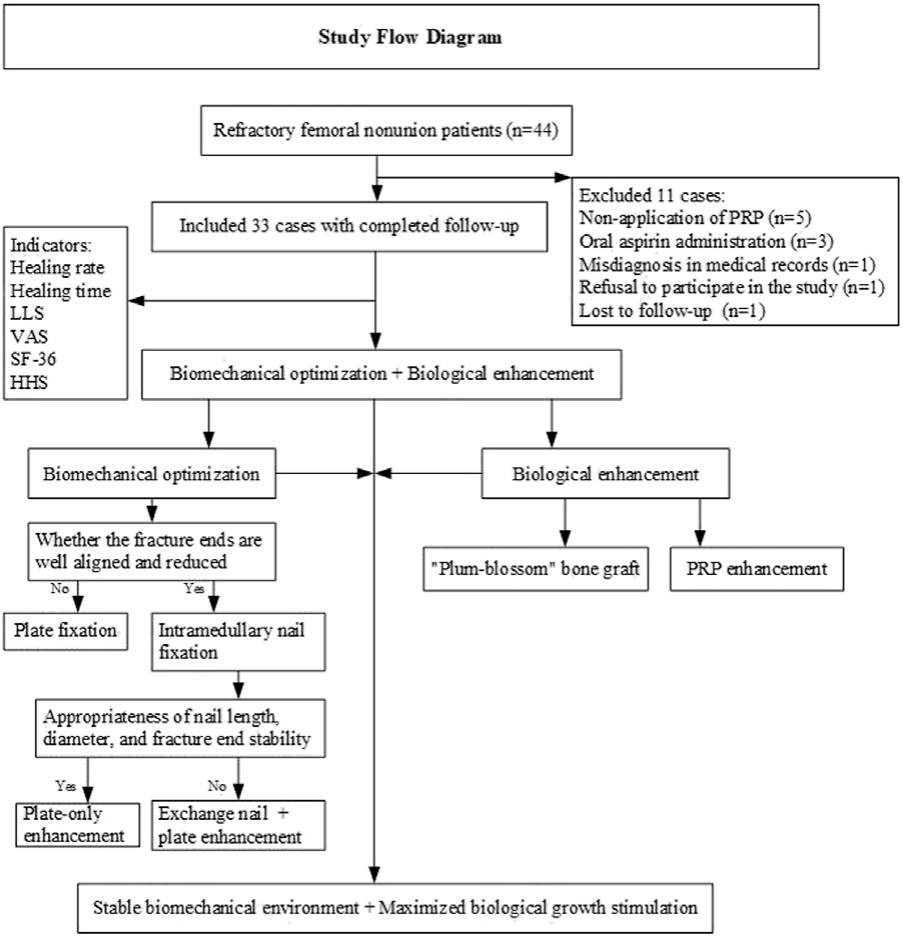


### Surgical technique

**FIGURE 1:**
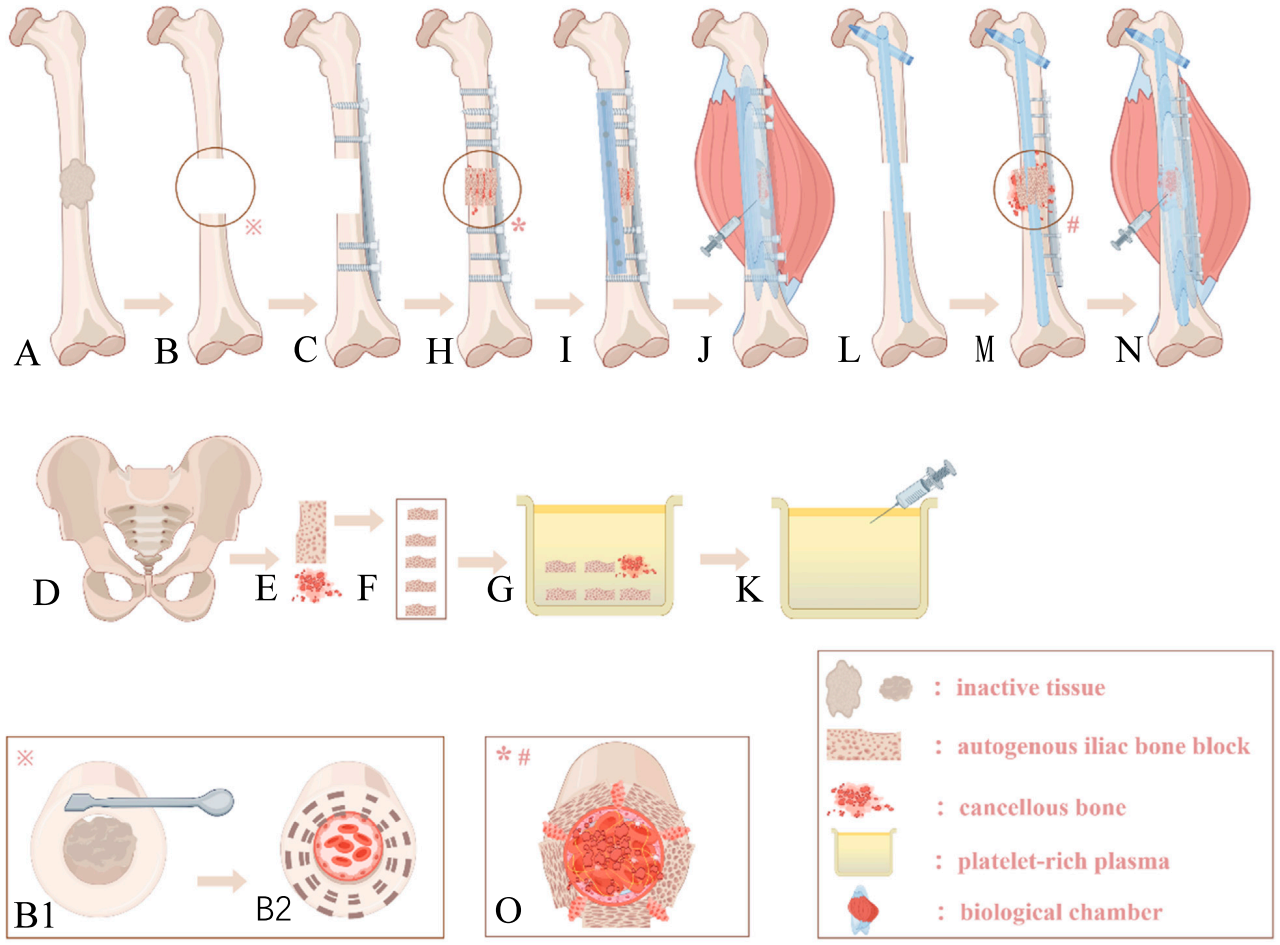
**(A)** The nonunion site is surrounded by non-viable tissue; **(B,B1,B2)** Thorough debridement of the nonunion site reveals the “red pepper” sign at the fracture ends; **(C)** A plate is inserted to maintain reduction after alignment correction; **(D,E)** Bone plates and cancellous bone are harvested according to the defect size; **(F)** The bone plates are trimmed into several cube-shaped blocks based on the defect morphology; **(G)** The trimmed bone blocks and cancellous bone are immersed in PRP; **(H)** The bone blocks are densely packed into the defect in a “plum blossom” pattern; **(I)** An auxiliary plate is placed on the anteromedial side; **(J,K)** The remaining PRP is injected into the bioactive chamber; **(L)** An intramedullary nail is inserted after alignment correction; **(M)** “Plum blossom” bone grafting with an auxiliary plate placed laterally; **(N)** Same as **(J,K)**; **(O)** A cross-sectional schematic of “plum blossom” bone grafting, showing cancellous bone implanted intramedullary, followed by tightly arranged cube-shaped bone blocks surrounding the defect, and cancellous bone packed into the gaps.

**FIGURE 2:**
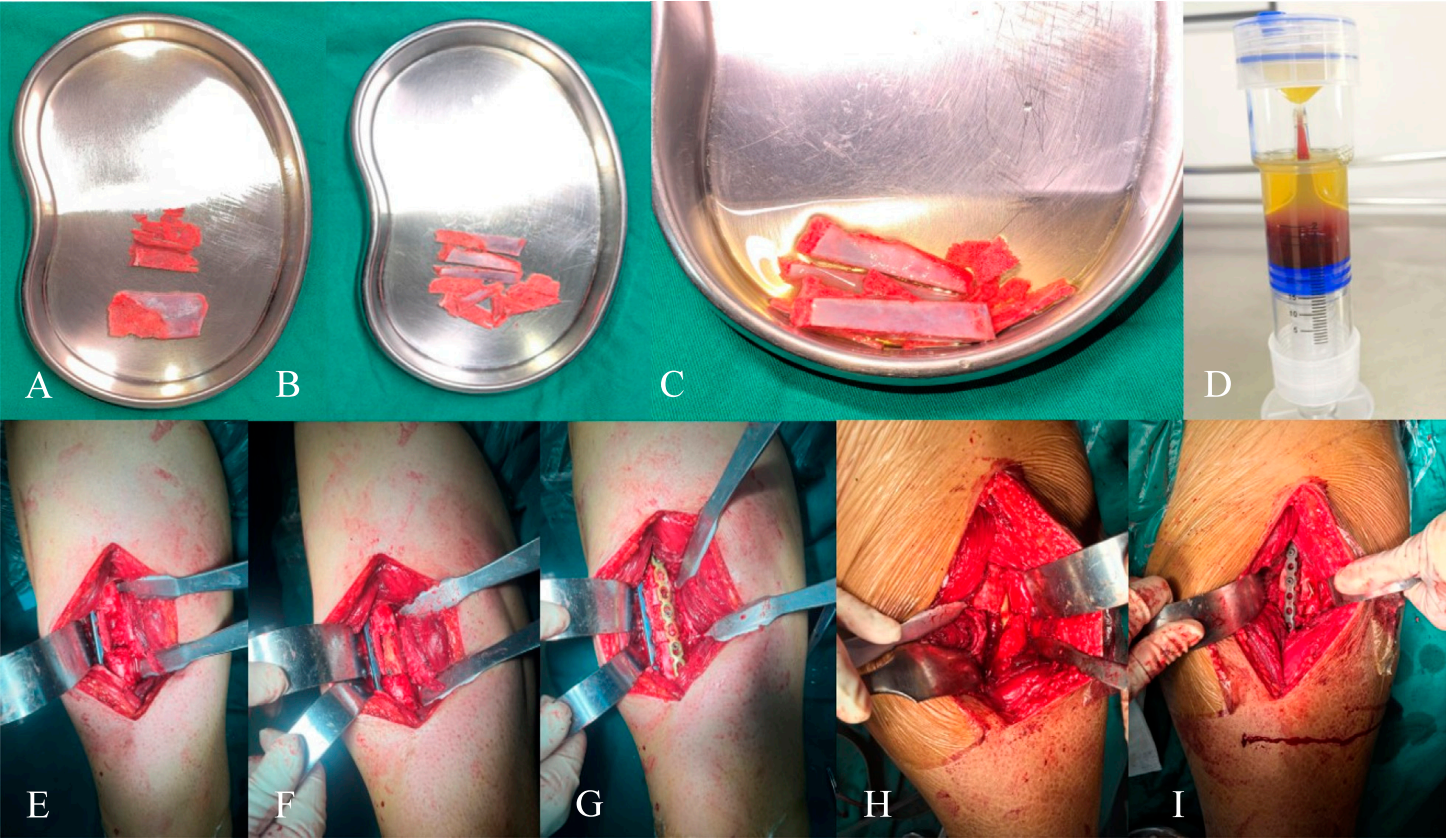
**(A)** Autologous iliac bone plates and cancellous bone; **(B)** The bone plates are trimmed into several cube-shaped blocks according to the defect morphology; **(C)** The modified bone blocks and cancellous bone are immersed in PRP; **(D)** Prepared PRP; **(E)** A plate is placed laterally to maintain reduction after alignment correction; **(F)** Cancellous bone is implanted intramedullary, and full-thickness bone blocks are densely arranged in a “plum blossom” pattern extramedullary; **(G)** After packing cancellous bone into the gaps, an auxiliary plate is placed on the anteromedial side; **(H)** An intramedullary nail is inserted after debridement and alignment correction, revealing the femoral defect intraoperatively; **(I)** After intramedullary implantation of cancellous bone, full-thickness bone blocks are sequentially placed around the intramedullary nail, followed by an auxiliary plate placed anterolaterally.

All surgeries were performed by four senior trauma orthopedic teams. Under anesthesia, patients were placed in the supine position, and the nonunion site was exposed through the previous incision. Failed internal fixation devices were removed, and non-viable tissue was debrided (Figure 1A) and sent for pathological examination. A fresh, bleeding bone bed was prepared (Figures 1B,B1,B2), followed by pulsed lavage (containing clindamycin 900 mg/L and tobramycin 80 mg/L). Due to previous bone harvesting, the Trapdoor minimally invasive technique (preserving the medial cortex) was used to minimize donor site morbidity, with graft volume adjusted based on defect size (Figures 1D,E, 2A). Fifty milliliters of blood was drawn from the patient’s elbow vein and added to a PRP tube (Weigao, China) preloaded with 5 mL of sodium citrate reagent. The tube was placed in a centrifuge (Weigao, China) and centrifuged at 2,000 r/min for 10 min. Ten milliliters of the pale yellow supernatant in the upper layer was aspirated, sampled for count detection. The detected PRP concentration was 4–5 times the original count (range: 700 × 10^9/L∼900 × 10^9/L). It should be noted that the prepared PRP is of the leukocyte-rich type (Figure 2D). The iliac graft was trimmed into 3–5 cubic bone blocks (Figures 1F, 2B) and soaked in PRP with cancellous bone particles (Figures 1G, 2C). Remaining PRP was stored at 4 °C in a sterile incubator. Based on intraoperative needs and surgeon preference, dual-plate fixation or intramedullary nailing with plating was performed.

Dual-Plate Fixation: After debridement and irrigation, alignment was corrected, and the defect was documented. A limited-contact locking compression plate was placed on the lateral femur (Figures 1C, 2E). PRP-enhanced bone blocks were arranged in a “plum blossom” pattern and tightly packed into the defect (Figures 1H, 2F), with cancellous bone particles filling the medullary cavity and gaps (Figure 1O). After compression, appropriate screws were inserted, and a second plate was placed on the anteromedial femur for compression and bicortical fixation (Figures 1I, 2G). Intramedullary Nailing with Plating: After debridement and irrigation, a larger intramedullary nail was inserted (Figures 1L, 2H). Bone debris from reaming was mixed with PRP-enhanced iliac bone blocks for “plum blossom” grafting (Figure 1O). A plate was then placed on the lateral femur (Figures 1M, 2I). After hemostasis, absorbable sutures were used to create a sealed chamber around the fracture site, and the remaining PRP solution was injected (Figures 1J,K,N). The incision was closed in layers, and routine compression dressing was applied.

### Postoperative management

All patients received systematic preoperative smoking cessation counseling and education on fracture nonunion. Second-generation cephalosporins were administered prophylactically for 48 h postoperatively. Rehabilitation training, including quadriceps exercises and ankle pump exercises, began on the first postoperative day. Partial weight-bearing with a walker was initiated within 2–4 weeks postoperatively, with gradual weight-bearing progression based on clinical and radiographic improvement. Oral calcium and vitamin D supplements were prescribed for 1 month after discharge. Follow-up visits were conducted at 1, 2, 3, 6, 9, and 12 months postoperatively, with X-rays taken at each visit to assess healing (independently evaluated by a senior trauma specialist and two radiologists unrelated to the study). At the final follow-up, visual analog scale (VAS) pain score (Åström et al., 2023), SF-36 quality of life score (Grönkvist et al., 2024), Harris hip score (HHS) (Cho et al., 2025), and limb shortening (assessed by a senior orthopedic surgery resident) were evaluated.

### Data collection and statistical analysis

Demographic data (age, gender, etc.), baseline characteristics (smoking status, disease duration, nonunion type, etc.), disease parameters (limb shortening, femoral defect size, etc.), efficacy outcomes, and complications were retrospectively analyzed. Continuous data were expressed as mean ± standard deviation, and categorical data were presented as frequencies and percentages. Since no control group was established, only pre- and post-treatment data from the single group were compared for differences. The Shapiro-Wilk test was used to assess normality for continuous variables. Paired t-tests were used for normally distributed variables, and the Wilcoxon signed-rank test was used for non-normally distributed variables. Data analysis was performed using SPSS (version 26.0; IBM Corp., Armonk, NY, USA), with all tests being two-tailed and a significance level set at P < 0.05.

## Results

Of 293 initially screened femoral nonunion cases, 44 met the refractory criteria. Eleven cases were excluded (5 did not receive PRP therapy, 3 were on aspirin, 1 had medical record errors, 1 declined participation, and 1 had incomplete follow-up data), leaving 33 patients for analysis. All patients completed follow-up, with a mean duration of 16.06 ± 3.37 months (range: 12–24 months). There were 24 males and 9 females, with a mean age of 42.64 ± 13.03 years (range: 18–71 years) and a mean of 2.64 ± 1.17 previous surgeries (range: 2–6, excluding the current revision). Nonunion types included hypertrophic (4 cases, 12.10%), atrophic (24 cases, 72.70%), and oligotrophic (5 cases, 15.20%). The locations were femoral shaft (24 cases, 72.70%) and supracondylar femur (9 cases, 27.30%), with a mean defect size of 3.87 ± 1.05 cm (range: 1.8–6 cm). Detailed demographic, baseline, and disease data are shown in Table 1.

**TABLE 1:** Demographic information and disease parameters of 33 patients.

Variable	Outcome	
Gender		
Female	9	(27.30%)
Male	24	(72.70%)
Age(years)	42.64 ± 13.03	(18–71)
BMI(kg/m^2^)	24.66 ± 1.55	(21.16–27.55)
Smoking history		
Yes/No	11	(33.70%)
Blood loss (mL)	451.52 ± 317.83	(150–1100)
Hospital stay (days)	12.58 ± 5.60	(5–32)
Number of surgeries	2.64 ± 1.17	(2–6)
Time from initial surgery to current revision (months)	23.35 ± 5.47	(19–48)
Follow-up duration (months)	16.06 ± 3.37	(12–24)
Femoral defect (cm)	3.87 ± 1.05	(1.8–6)
Anatomical location		
Femoral shaft	24	(72.70%)
Supracondylar femur	9	(27.30%)
Nonunion type		
Hypertrophic	4	(12.10%)
Atrophic	24	(72.70%)
Oligotrophic	5	(15.20%)
Injury mechanism		
Traffic accident	21	(63.60%)
Fall	12	(36.40%)
Side		
Left	16	(48.50%)
Right	17	(51.50%)
Pre-revision fixation		
INAP	20	(60.60%)
DP	13	(39.40%)
Post-revision fixation		
EINAP	14	(42.40%)
DP	19	(57.60%)

Categorical data are presented as counts and percentages; continuous data are presented as mean ± standard deviation and range. INAP: intramedullary nail + adjuvant plate. EINAP: Exchange intramedullary nail + adjuvant plate DP: double plates.

The overall union rate was 96.97% (32/33), with a mean healing time of 9.78 ± 1.75 months (range: 7.00–13.50 months). The mean VAS score improved significantly from 4.27 ± 1.18 (range: 1–6) preoperatively to 1.21 ± 1.05 (range: 0–5) postoperatively (P < 0.001). The mean HHS increased from 50.91 ± 8.47 (range: 38–65) to 86.39 ± 7.75 (range: 49–94) (P < 0.001). The mean SF-36 score improved from 59.21 ± 5.63 (range: 45–70) to 84.48 ± 5.32 (range: 60–93) (P < 0.001). Limb shortening decreased from 2.20 ± 0.64 cm (range: 0.90–3.50) to 0.32 ± 0.57 cm (range: 0–2.50) (P < 0.001). Detailed results are shown in Table 2.

**TABLE 2:** Analysis of treatment efficacy differences before and after treatment in 33 patients.

Evaluation indicators	Pre-revision	Final follow-up	Statistic	P-value
VAS	4.27 ± 1.18	1.21 ± 1.05	*Z* = −5.020	**<0.001**
HHS	50.91 ± 8.47	86.39 ± 7.75	*Z* = −5.014	**<0.001**
SF-36	59.21 ± 5.63	84.48 ± 5.32	*Z* = −4.997	**<0.001**
LLS (cm)	2.20 ± 0.64	0.32 ± 0.57	*Z* = −5.015	**<0.001**
Healing status (union/nonunion)	—	32/1	—	—

P-values <0.05 are bolded. Continuous variables are expressed as mean ± standard deviation. VAS: visual analogue scale; HHS: harris hip scoring system; SF-36: SF-36 Quality of Life Scale. LLS: Lower limb shortening. *Z*: standardized test-statistic.

Subgroup analysis after excluding the one nonunion case showed that functional improvements remained statistically significant (Table 3). Among the 33 patients, 11 (33.33%) experienced postoperative limb shortening, with 2 (6.10%) affecting function. After excluding the nonunion case, 9 (28.13%) had limb shortening, with 1 (3.12%, shortening of 1.5 cm) affecting function. Based on medical records and history, 2 (6.06%) had severe complications: 1 (3.03%) persistent nonunion and 1 (3.03%) deep vein thrombosis. No cases of heterotopic ossification, infection, or immune rejection were observed.

**TABLE 3:** Analysis of treatment efficacy differences before and after treatment in 32 patients.

Evaluation indicators	Pre-revision	Final follow-up	Statistic	P-value
VAS	4.22 ± 1.16	1.09 ± 0.82	*Z* = −4.950	**<0.001**
HHS	51.19 ± 8.45	87.56 ± 3.93	*t* = −22.249,95%CI (−39.71, −33.04)	**<0.001**
SF-36	59.00 ± 5.58	85.25 ± 3.05	*t* = −22.452,95%CI (−28.64, −23.87)	**<0.001**
LLS (cm)	2.16 ± 0.61	0.25 ± 0.42	*Z* = −4.940	**<0.001**
Mean time to union (months)		9.78 ± 1.75		

P-values <0.05 are bolded. Continuous variables are expressed as mean ± standard deviation. VAS: visual analogue scale; HHS: harris hip scoring system; SF-36: SF-36 Quality of Life Scale. LLS: Lower limb shortening. t: t-statistic; *Z*: standardized test-statistic.

## Discussion

**FIGURE 3:**
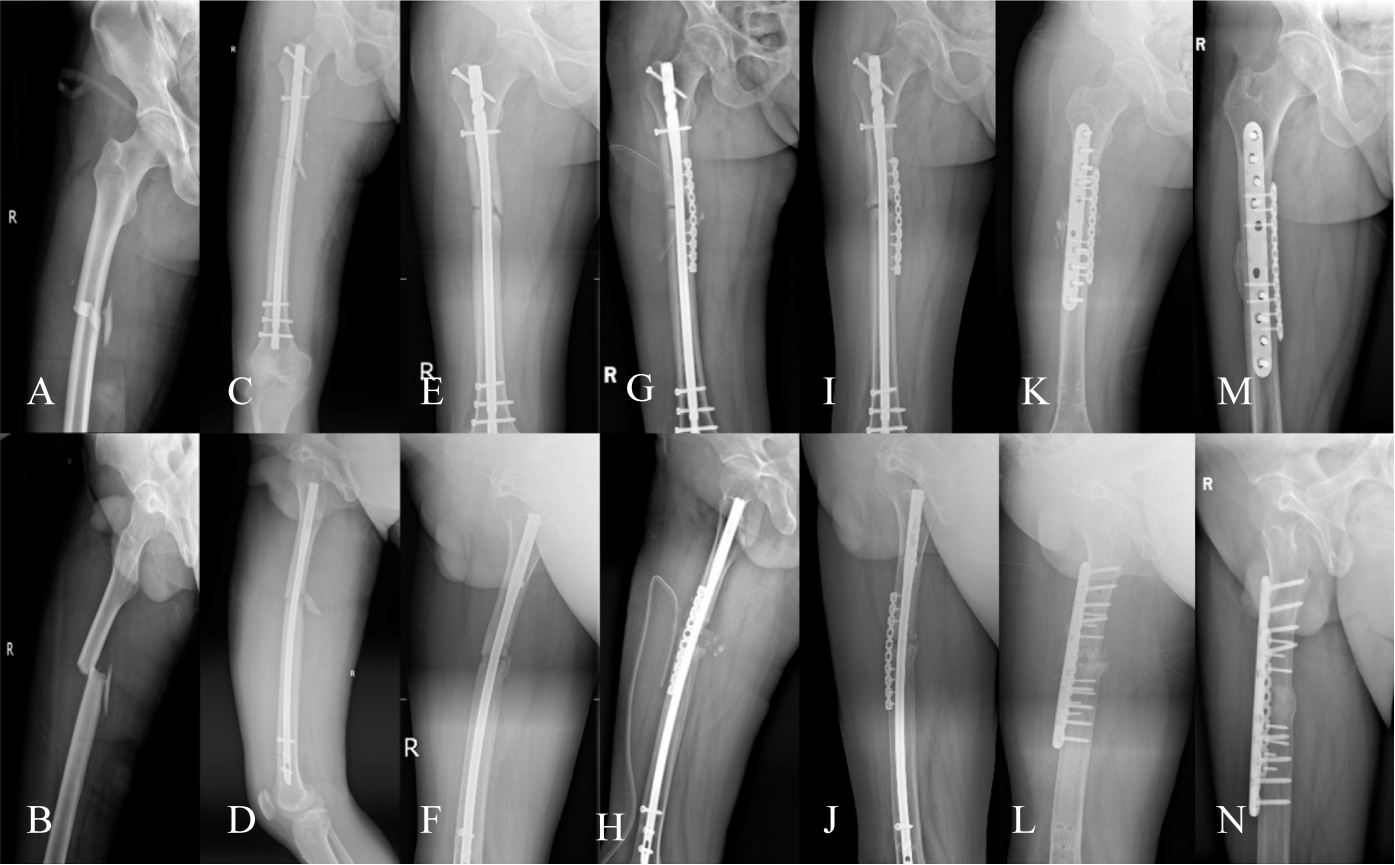
A 39-year-old male, BMI: 27.13 kg/m^2^, with no smoking history or metabolic diseases. **(A,B)** A car accident on 5 January 2021, resulted in a right femoral shaft fracture; **(C,D)** Closed reduction and intramedullary nail fixation were performed on 13 January 2021; **(E,F)** Follow-up on 8 October 2021, revealed nonunion with minimal callus formation; **(G,H)** A nonunion revision surgery was performed on 23 October 2021, without replacing the intramedullary nail, using autologous iliac cancellous bone grafting and auxiliary plate augmentation; **(I,J)** Follow-up on 7 July 2022, showed persistent nonunion; **(K,L)** A second nonunion revision surgery was performed on 12 July 2022, using dual locking plate fixation, PRP-enhanced “plum blossom” autologous iliac bone grafting, and the construction of a bioactive center; **(M,N)** Follow-up at 9.5 months postoperatively demonstrated excellent bony union, with an SF-36 score of 87, HHS score of 89, and VAS score of 1.

**FIGURE 4:**
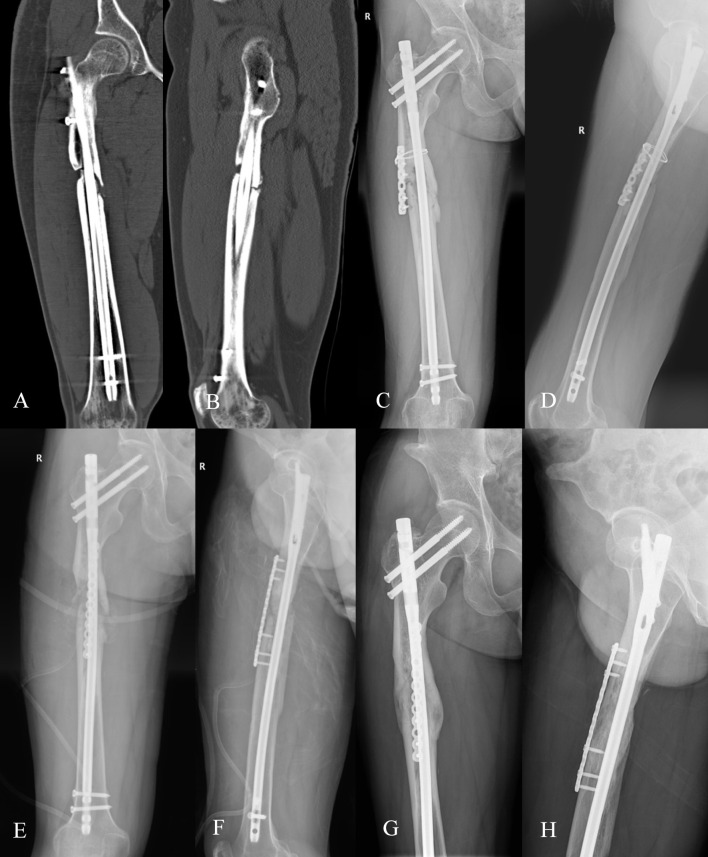
A 51-year-old male patient with a body mass index of 25.60 kg/m^2^ and a 15-year smoking history. In September 2020, he sustained a right femoral shaft fracture due to a traffic accident and underwent closed reduction and intramedullary nailing at another hospital; a re-examination 10 months postoperatively revealed fracture nonunion, and he underwent the first revision surgery (nail exchange + auxiliary plate internal fixation + autologous iliac bone grafting) at the original hospital in August 2021; a re-examination in December 2022 still showed fracture nonunion, and he was subsequently referred to our hospital. **(A,B)** X-ray films before the first revision surgery (9 August 2021), showing right femoral shaft fracture nonunion with bone resorption at the fracture end; **(C,D)** X-ray films 16 months after the first revision surgery (15 December 2022), showing persistent fracture nonunion and backout of the distal screws of the auxiliary plate; **(E,F)** X-ray films after the second revision surgery (22 December 2022), surgical method: auxiliary plate exchange + platelet-rich plasma (PRP) combined with autologous iliac bone grafting; **(G,H)** Outpatient follow-up X-ray films 10 months postoperatively (19 October 2023), showing satisfactory fracture healing; functional scores: SF-36 score 81 points, Harris Hip Score 91 points, Visual Analog Scale score 0 points.

Initial fracture nonunion often achieves satisfactory outcomes with biomechanical optimization and autologous bone grafting (Siverino et al., 2024; Wildemann et al., 2021)^.^ However, some cases refractory to standard treatment progress to recalcitrant nonunion, which is multifactorial and disrupts the “bone healing unit” environment (Nicholson et al., 2021). In this study, 87.90% of refractory nonunions were of low biological activity, indicating poor healing potential. Studies (Elliott et al., 2016) suggest that biologically derived nonunions require additional measures to enhance the “bone healing unit,” such as bone morphogenetic protein (BMP), stem cells, demineralized bone matrix (DBM), and platelet-rich plasma (PRP) (Gagnon et al., 2024; Siverino et al., 2024). This study optimized the local biological environment on a stable biomechanical foundation: “plum blossom” bone grafting increased the contact area between the graft and bone bed, intramedullary cancellous bone and extramedullary plates facilitated endosteal and periosteal osteoinduction, PRP enhanced graft activity, and a bioactive chamber encapsulated the nonunion site, creating a sustained-release “bone healing center” that reversed the nonunion process, achieving healing in cases with multiple failed revisions (overall union rate: 96.97%). Typical cases are illustrated in Figures 3, 4.

### Application of PRP in fracture nonunion

PRP is a concentrated platelet suspension derived from autologous venous blood, offering high biosafety and avoiding risks of immune rejection and disease transmission (Farshidfar et al., 2025; Shi et al., 2025). In addition to high platelet concentration, PRP contains various bioactive factors, such as platelet-derived growth factor (PDGF), transforming growth factor-β (TGF-β1, TGF-β2), vascular endothelial growth factor (VEGF), epidermal growth factor (EGF), insulin-like growth factor (IGF-1), and fibrin (Farshidfar et al., 2025; Shi et al., 2025). These factors directly or indirectly activate mesenchymal stem cell osteogenic differentiation, promote vascularization, optimize the microenvironment, exert anti-inflammatory and immunomodulatory effects, and enhance bone matrix synthesis and mineralization, thereby facilitating bone repair (Chang et al., 2025; Ding et al., 2019; Farshidfar et al., 2025; Liebig et al., 2020; Shi et al., 2025). Wittig et al. (2016) reported a 100% union rate in a study of refractory nonunions (tibia and femur, n = 3) using autologous bone marrow-derived mesenchymal stem cells (MSCs) expanded *in vitro* and loaded with PRP gel onto collagen microspheres implanted at the nonunion site. MSCs possess osteogenic, chondrogenic, adipogenic, and endothelial differentiation capabilities, while PRP promotes MSC proliferation and differentiation. Collagen microspheres act as carriers for sustained growth factor release, providing stable biological stimulation. This hybrid approach was reported and recommended by the American Academy of Orthopaedic Surgeons (AAOS) (Wellings et al., 2024). Compared to Wittig et al. (2016), our surgical procedure was simpler (no *in vitro* culture required) and involved a larger case series. Bettega et al. (Bettega et al., 2009) demonstrated in a retrospective controlled trial of 18 bone defect cases that PRP combined with minimal autograft reduced bone graft volume by approximately 60% (P < 0.05), consistent with findings by Dohle et al. (2024) and Lee et al. (2009) on PRP’s synergistic mechanisms. Choi et al. (2024) mixed biological agents with hydroxyapatite particles and autologous cancellous bone, and implanted the mixture into the defect area. Hydroxyapatite particles and autologous cancellous bone are rich in porous structures, which can fully adsorb and load BMP. Additionally, Choi et al. constructed a creeping template for bone growth through soft tissue. This approach not only prevented the leakage of BMP but also induced the formation of bones with normal anatomical morphology. Similarly, Nie et al. (2021) mixed PRP with DBM into a “cement-like” substance, which enhanced biological activity while preventing PRP leakage. Furthermore, they adopted soft tissue coverage technology to protect the graft. In this study, soaking the graft in PRP allowed the porous cancellous bone to absorb and load growth factors, enhancing osteogenic activity and providing sustained biological stimulation post-implantation. Recent reviews (Bacevich et al., 2024) emphasize the need for controlled, sustained growth factor release to match the bone regeneration process, highlighting the advantages of sustained-release strategies.

### Role of the bioactive chamber

Research indicates (Calori and Giannoudis, 2011) that soft tissue, periosteum, cortical bone, and bone marrow at the injury site collectively contribute to healing, with their roles influenced by factors such as mechanical stability of the callus, growth factor levels, oxygen tension, and hormones. The bioactive chamber developed in this study incorporates an osteoconductive scaffold (iliac bone graft), mesenchymal stem cells (bone marrow and marrow blood), and growth factors (PRP), forming a bioactive center. This localized “bioreactor” exhibits anti-inflammatory properties (a randomized controlled trial by Zitsch et al. (2022) demonstrated that PRP reduces IL-8, PGE2, and IL-1RA levels); its enclosed structure contains and protects PRP, preventing leakage and providing physical protection; studies by Fan et al. (2024) and Nie et al. (2021) suggest it promotes tissue and wound repair, enhancing healing. Similar to the studies by Choi et al. (2024), Nie et al. (2021), and Chamseddine et al. (2022), we also adopted soft tissue coverage protection technology (to construct a bioactive chamber). After adequate hemostasis, the remaining active tissues around the fracture site (such as periosteum and fascia) were sutured into a closed chamber surrounding the fracture ends, and PRP solution was injected into this chamber (Figures 1J,N). We believe this approach can achieve slow and continuous biological growth stimulation in the early stage of bone healing and enhance the biological potential of the “bone healing unit”. This design aligns with the “biological chamber effect of fracture healing” reported by Calori and Giannoudis (2011) and is central to the “diamond concept,” aiding in the treatment of refractory atrophic nonunion.

### Importance of mechanical stability

Mechanical stability is a critical factor for achieving union (Elliott et al., 2016; Nicholson et al., 2021; Siverino et al., 2024; Wildemann et al., 2021). Excessive strain at the fracture site disrupts the “bone healing unit,” forming a synovial cavity and hindering bone end healing. Therefore, revision surgery requires selecting an appropriate internal fixation system to reduce strain at the fracture site to within the healing threshold. El-Alfy et al. (2024) achieved a 96.15% union rate in 26 cases of refractory subtrochanteric nonunion using “lateral closing wedge valgus reduction + valgus-shaped dynamic compression screws” to reduce strain at the nonunion site, combined with “decortication and bone grafting” to improve the local biological environment. Özdemir et al. (2022) improved the local biological environment in 7 cases of refractory upper limb nonunion using vascularized medial femoral condyle bone grafts on a stable mechanical foundation. Compared to these studies, our focus on refractory femoral shaft and supracondylar nonunions employed simpler mechanical reconstruction and biological enhancement techniques.

In this study, 42.40% of cases underwent intramedullary nail replacement combined with auxiliary plate augmentation. Replacing with a larger intramedullary nail provides greater mechanical load-bearing advantages and activates biological effects through reaming, while the auxiliary plate compensates for the nail’s rotational stability limitations, transitioning the mechanical structure from elastic to rigid fixation. Biomechanical studies (Park et al., 2011; Walcher et al., 2023) show that this combined approach offers 3.3 times greater bending and torsional resistance than intramedullary nails alone; clinical studies (Devendra et al., 2024; Kook et al., 2024) confirm that intramedullary nails combined with auxiliary plates yield higher success rates than nail replacement alone. However, this nail-oriented strategy faces limitations in graft space and anatomical constraints (Brinker and O'Connor, 2007; Kook et al., 2024). In this study, the innovative use of PRP-enhanced “plum blossom” bone grafting technique, where iliac bone was trimmed into 3-5 petal-shaped blocks to precisely fit the defect morphology, with cancellous bone filling the gaps and auxiliary plate reinforcement, addressed these limitations. This design compensates for the restricted graft space due to the nail’s presence and achieves simultaneous intramedullary and extramedullary osteogenesis through the cancellous bone-plate system, combining mechanical and biological advantages (Brinker and O'Connor, 2007; Devendra et al., 2024; Kook et al., 2024). Dual-plate systems, with their superior rotational stability (especially in non-isthmus fractures), serve as an important alternative (Gavaskar et al., 2024; Taheriazam et al., 2024; Wang et al., 2022). Wang et al. (2022) used dual-plate fixation and created a 4 cm × 1 cm bone groove for graft implantation in 186 femoral nonunion cases, enhancing osteogenic potential but raising concerns about excessive bone stripping in atrophic nonunion treatment (Andersen et al., 2021). In contrast, our approach optimized graft morphology to preserve host bone structure maximally.

In this study, mechanical reconstruction reduced strain, providing a stable mechanical environment for healing, while PRP-enhanced “plum blossom” autologous iliac bone grafting and the bioactive chamber maximized biological growth stimulation, promoting bone healing and remodeling, enabling earlier functional rehabilitation. Accelerated healing through reduced disability duration helps minimize complications and improve overall outcomes. Functional assessment data showed an average Harris hip score (HHS) of 86.39, SF-36 score of 84.48, and pain score as low as 1.21, confirming the treatment’s positive impact on quality of life. Notably, functional improvements stemmed not only from fracture healing but also from preoperative and postoperative health education, psychological support, and professional rehabilitation training, combined with patient cooperation, accelerating limb functional recovery.

Bone transport techniques (e.g., Ilizarov technique) and membrane-induced techniques (e.g., Masquelet technique) are effective methods for treating refractory nonunion, especially for those complicated with segmental defects or infections (Nicholson et al., 2021; Wildemann et al., 2021). The Ilizarov technique promotes bone regeneration through continuous tension-stress effect. Compared with plate internal fixation, it has advantages of minimal stress shielding effect and relatively limited damage to periosteal and soft tissue blood supply (Ding et al., 2021). However, this technique has a long treatment cycle, may require secondary surgery to handle the junction of the bone regeneration area (Ren et al., 2023), and the regenerated bone has low mechanical strength that needs a long time for bone remodeling (Li et al., 2022; Ren et al., 2023). Additionally, there is a risk of pin tract infection associated with the external fixator (Li et al., 2022; Ren et al., 2023; Tan et al., 2024). Nevertheless, it still holds an irreplaceable value in the treatment of large segmental bone defects and infectious nonunion (Ren et al., 2023). A key step of the Masquelet technique involves implanting materials to induce the formation of a bioactive membranous structure in the defect area, which usually proceeds in two stages: initial surgery to implant bone cement for inducing active membrane formation, followed by secondary surgery for bone grafting (Alford et al., 2021). Although effective for refractory nonunion, this method also faces challenges such as multiple surgeries, high surgical difficulty, and a prolonged treatment cycle (Alford et al., 2021). Compared with the aforementioned techniques, the one-stage surgical protocol adopted in this study shows obvious advantages in shortening the treatment cycle and improving patient compliance. Our technique is modified based on mature bone grafting and internal fixation: optimizing the osteoconductive structure through “plum-blossom shaped” bone grafting, enhancing biological activity by combining with PRP, and constructing a closed bioactive chamber to maintain a local environment rich in growth factors. This protocol has a clear technical route and good clinical operability while achieving the dual goals of biological and mechanical repair.

## Limitations

This study has the following limitations: 1. Limited literature on refractory femoral fracture nonunion restricts comparative analysis; 2. Retrospective design introduces selection bias (despite independent tripartite evaluation); 3. Mixed internal fixation methods may confound result interpretation; 4. This study did not include a control group, which constitutes a major limitation. Due to the extremely low incidence of refractory femoral nonunion with failed revisions and ethical considerations that preclude the reapplication of previously unsuccessful traditional treatment regimens, it was challenging to recruit control samples with consistent baseline characteristics. The results of this study are primarily applicable to such highly complex patients and cannot be directly extrapolated to populations with conventional bone nonunion. Future research aims to conduct prospective multicenter studies to expand the sample size and perform further comparative analyses. 5. The bone defect size included in this study did not exceed 6 cm, and the clinical efficacy for segmental bone defects larger than 6 cm remains uncertain. Future studies should control for internal fixation types, include a control group to validate PRP’s biological effects, or conduct multicenter prospective randomized controlled trials.

## Conclusion

Nonunion cases refractory to autologous iliac bone grafting require additional interventions. In this study, optimizing bone grafting methods and using the biological agent—platelet-rich plasma—enhanced therapy yielded excellent outcomes. Furthermore, this study provides a simple, efficient, and replicable technique for the clinical treatment of refractory and recalcitrant femoral nonunion.

## Data Availability

The raw data supporting the conclusions of this article will be made available by the authors, without undue reservation.
